# The mechanism debate in psilocybin therapy for depression

**DOI:** 10.1016/j.ebiom.2026.106440

**Published:** 2026-08-12

**Authors:** eBioMedicine

The use of psilocybin for treatment-resistant depression (TRD) is moving towards clinical use, and two unresolved questions will shape what comes next: how large and durable the benefit is, and what produces it? The strongest peer-reviewed efficacy signal is a phase 2b trial, in which one dose of synthetic psilocybin with psychological support approximately doubled the depression-score reduction at 3 weeks versus a low-dose control, although the benefit was unclear by 12 weeks. Potentially pivotal phase 3 results remain topline company announcements; however, these have not yet been peer reviewed. Compass Pathways' COMP005, the first phase 3 trial of a classic psychedelic, claims to have met its primary endpoint in TRD; the larger COMP006 reports a durable response up to 26 weeks. However, the effect is modest: only a minority of patients reached a clinically meaningful response. Early observational cohorts show lower response and remission rates than previous trials.

Understanding the mechanism of action is where the field is most divided. One proposal, based largely on preclinical work, suggests that psilocybin works by promoting neuroplasticity and that this can be separated from its psychedelic experience. This premise underlies non-hallucinogenic neuroplastogens entering clinical trials. Delix Therapeutics’ zalsupindole is proposed to modulate neuroplasticity markers without hallucinogenic or dissociative effects and has reported early antidepressant signals in phase 1b (unpublished company data), with an FDA-cleared at-home phase 2 to follow. However, this clean separation of neuroplasticity from experience has been shown mainly in animals; in humans it is largely unproven. Ketamine offers the closest, albeit imperfect, clinical precedent: in a dose-ranging trial, doses with the clearest antidepressant effects also produced the most dissociation, and dissociation intensity did not correlate with symptom improvement, leaving unresolved whether the experience contributes to or merely accompanies antidepressant action. The competing explanation holds that the experience itself is the mechanism: psychological insight, emotional breakthrough, and the quality of the altered state carry the effect. In some studies, intensity of the acute experience tracks lasting improvement in depressive symptoms. However, findings vary and the link weakens as masking improves, leaving open whether the experience drives recovery or merely reflects expectancy.

Imaging studies do not resolve this divide. In healthy volunteers receiving their first psilocybin dose, acute electroencephalogram (EEG) signal complexity (cortical entropy) predicted next-day psychological insight and 1-month wellbeing, with insight mediating the link—preliminary evidence that the experience is part of the mechanism, not a by-product. A parallel structural change—apparent compacting of nerve fibres in prefrontal–subcortical circuits—might, if replicated, reflect neuroplasticity. But the marker is ambiguous: the same reduction accompanies learning, healthy development, and axonal injury. The experiential functional EEG signal proved more robust than the structural one—a notable result, given how much excitement and investment rests on the neuroplasticity story. Even so, the entropy biomarker is contested: an independent cohort found that only a minority of fMRI entropy metrics track psilocybin, and a critical review questioned whether brain entropy is specific to the psychedelic state.

Across both hypotheses sits a methodological problem: functional unmasking. When a drug's effects are unmistakable, participants guess their allocation and expectancy contaminates outcomes. A systematic review found unmasking rates above 90%, with most trials not assessing it. Under equal unmasking, a meta-analysis found that the advantage over antidepressants was substantially attenuated. Regulators have also noticed: the US Food and Drug Administration cited functional unmasking when rejecting Lykos Therapeutics' MDMA-assisted therapy for post-traumatic stress disorder in 2024, and its July, 2026 guidance codifies specific safeguards: active placebos, expectancy measurement, and dose–response designs. Expectancy is where the mechanistic and regulatory questions converge: if experience is part of the mechanism, unmasking is not just a bias to control but a clue to how treatment works.

Regulators and clinicians will scrutinise a modest, expectancy-sensitive effect of disputed mechanism. Non-hallucinogenic programmes will offer the first clue—if a plasticity-matched compound with no subjective effect proves durably effective, plasticity as a mechanism might prevail; if not, the experience could earn its place. More likely, neither wins outright: plasticity might open the window while the experience decides what passes through it. In mice, the duration of critical-period reopening scales with the drug's acute subjective effects—making their interaction the question worth researching. Progress will require designs that model expectancy rather than eliminate it and causal–mediation analyses studies in patients with TRD, not healthy volunteers. The stakes are not only scientific: if the experience does part of the work, a reimbursement model built for molecules alone will undervalue the psychological support that makes them effective. Whether psilocybin becomes a mainstream depression treatment will hinge less on proving that the brain changes than on pinpointing what, in a deliberately overwhelming experience, makes people well.

